# Oncological outcomes of selective axillary dissection with 4% carbon marking

**DOI:** 10.1590/0100-6991e-20243697-en

**Published:** 2024-11-07

**Authors:** LUCAS ROSKAMP BUDEL, CLEVERTON CÉSAR SPAUTZ, MARIA HELENA LOUVEIRA, TERESA CRISTINA SANTOS CAVALCANTI, ALESSANDRA CORDEIRO FORNAZARI, PLINIO GASPERIN, LEONARDO NISSEN, VINICIUS MILANI BUDEL

**Affiliations:** 1 - Universidade Federal do Paraná, Departamento de Tocoginecologia - Curitiba - PR - Brasil; 2 - Universidade Federal do Paraná, Departamento de Radiologia - Curitiba - PR - Brasil; 3 - Universidade Federal do Paraná, Departamento de Patologia - Curitiba - PR - Brasil

**Keywords:** Breast Neoplasms, Sentinel Lymph Node, Carbon, Neoplasm Staging, Neoadjuvant Therapy, Tratamento Neoadjuvante, Câncer de Mama, Biópsia do Linfonodo Sentinel, Cirurgia Axilar, Dissecção Axilar Seletiva

## Abstract

**Introduction:**

The use of axillary marking prior to Neoadjuvant Systemic Therapy (NST) is a controversial matter regarding patients with positive Lymph Nodes (LN). Several methods were tested to make possible the decrease of false negative rate in comparison to sentinel lymph node adding more accuracy to the results. This study aims to evaluate the oncological outcomes in patients who had undergone selective axillary dissection with 4% carbon marking before TSN.

**Methods:**

A prospective study was performed with cT1-T4, cN1-N2 breast cancer patients classified as suspected LNs undergoing concomitant 4% carbon marking. After TSN, targeted LNs were identified and resected associated to the sentinel lymph node (SLN) biopsy. The oncological outcomes pointed out were overall survival (OS), causespecific survival (CSS), distant disease-free survival (DDFS), axillary recurrence (AR) and local recurrence (LR).

**Results:**

A total of 168 patients were evaluated for a median period of 49 months. The axillary emptying was reached in 89 (50.6%) cases. Five of 168 patients (2.9%) had axillary recurrence (AR). There was a significant link between axillary emptying and AR (0 vs. 6% p = 0.012). The DDFS was 140/168 (83.3%), OS 158/168 (94%) and CSS 158/163 (96.9%).

**Conclusion:**

The use of carbon marking in selective axillary dissection is a reliable low-cost method with simple execution. Among the oncological outcomes AR may not be considered for post downstaging axillary evaluation analysis since it is a rare event and not necessarily related to OS or DDFS.

## RESUMO

**Introdução:**

O uso de marcadores na axila antes da terapia sistêmica neoadjuvante (TSN) é um tema controverso em mulheres com linfonodos (LN) acometidos. Diversos marcadores foram utilizados resultando em uma redução da taxa de falso negativo ao comparar com o linfonodo sentinela, melhorando a acurácia. Este estudo tem como propósito analisar os resultados oncológicos em mulheres que passaram por dissecção axilar seletiva com marcação prévia à terapia sistêmica neoadjuvante (TSN) usando carvão a 4% e sua relação com o LN sentinela.

**Métodos:**

Foram selecionadas pacientes diagnosticadas com carcinoma invasivo de mama, apresentando estágios clínicos T1-4 e N1-2 submetidas à análise de LNs suspeitos e marcação pré TSN com carvão a 4%. Após o tratamento neoadjuvante, os LNs indicados com carvão foram excisados em um mesmo procedimento com o linfonodo sentinela. Os desfechos sobrevida global (SG), sobrevida específica (SE), sobrevida livre de doença a distância (SLDD), recidiva axilar (RA) e recidiva local (RL) foram catalogados.

**Resultados:**

Para a análise entre linfonodos marcados e linfonodo sentinela 176 pacientes foram incluídas com taxa de identificação de 95,4% do LN marcado. Para os desfechos oncológicos, cento e sessenta e oito pacientes foram analisadas em 49 meses de tempo mediano. O esvaziamento axilar foi omitido em 89 (50,6%) casos. Cinco/168 (2,9%) tiveram RA. Houve relação entre linfadenectomia axilar e RA (0 vs. 6% p=0,012). A SLDD foi 140/168 (83,3%), SG 158/168 (94%) e SE 158/163 (96,9%).

**Conclusões:**

O carvão se destaca como um marcador confiável, acessível e prático para dissecção axilar seletiva. O evento RA não deve ser utilizada para análise após TSN uma vez que é um evento raro e não está relacionada a SG ou SLDD diretamente.

## INTRODUCTION

Axillary examinations prior to neoadjuvant systemic therapy (NST) are a useful tool for staging and conducting the treatment of breast carcinoma^1^ ^-^ ^3^. However, after clinical response in previously compromised lymph nodes, there were uncertainties that limited the use of sentinel lymph nodes (SLN) due to its high false negative rate and low identification rate^4^ ^,^ ^5^. Tactics such as increasing the number of biopsied lymph nodes, applying immunohistochemistry, and using two ways to map the sentinel lymph node have rendered a false negative rate of less than 10%^6^ ^-^ ^8^. In these studies, all patients underwent lymphadenectomy, and it was not possible to evaluate the oncological outcomes in the preservation setting, and did not provide data on axillary recurrence (AR).

AR in women who had their axillary status reduced from positive to negative after NST is between 1.4 - 2.6%^9^ ^,^ ^10^. However, the evaluation of this outcome is not sufficient in the face of the new demand to lower false negative rates with the introduction of adjuvant drugs when complete pathological responses are not obtained^11^ ^-^ ^13^.

Applying techniques to mark a suspicious lymph node before NST and excising it afterwards increases the reliability of axillary evaluation without the need for lymphadenectomy, with false negative rates ranging from 1.4 to 4.2%^14^ ^-^ ^16^. Some of the barriers to the use of metal clips in these methods are cost, the need for interventional radiology before surgery, and the possibility of permanence of the clip after surgery in few patients^17^ ^-^ ^20^. In search of new, cheaper methodologies or with less technical difficulty, other materials were used for lymph node marking, such as magseed^®21^, Radar/Infrared^22^, clips visible on ultrasound14, and lymph node pigmentation through ink or carbon^23^ ^-^ ^25^.

Carbon has been used as a marker for non-palpable lesions in the breast for a long time^26^ ^,^ ^27^ and has been efficient in pre-NST lymph node marking, with lymph node identification rates between 96.4 and 100%^23^ ^-^ ^25^. The advantages reported in this technique include the ease of intraoperative identification and the non-need for an invasive localization procedure, thus reducing the burden on the patient by avoiding the use of radioactive materials.

This analysis aims to observe the oncological results in women undergoing selective axillary dissection in association with the sentinel lymph node and to evaluate its safety, as well as to evaluate the relationship between the sentinel lymph node and the marked lymph node prior to systemic therapy.

## METHODS

This study is a prospective cohort of longitudinal nature, conducted with a single group of patients who underwent selective axillary dissection combined with SLN biopsy, in the context of invasive breast carcinoma with clinical evidence of axillary involvement and who underwent neoadjuvant therapy. The procedures were performed in both public and private health facilities located in Curitiba, Paraná, during the period from July 2014 to January 2019. 

We selected patients diagnosed with invasive breast carcinoma confirmed by anatomopathological analysis, with clinical stages T1-4 and N1-2. We excluded from the study patients who had distant metastases at the time of diagnosis, histology other than invasive breast carcinoma, history of surgical breast biopsy and/or previous axillary surgical procedures, history of other malignant neoplasms, clinical contraindications to radiotherapy, inflammatory carcinomas, and inability to adhere to regular medical follow-up.

Since this was an analysis of axillary evaluation in patients with suspicious axillary lymph nodes, we also excluded cases of breast carcinoma with clinically negative axillae. Thus, there were no cases of T1 N0 breast cancer. As SLN is contraindicated in cases of inflammatory carcinoma, patients with this clinical presentation also did not participate in the study. 

The information collected was organized in Excel® spreadsheets, 2020 version. Quantitative variables were summarized using measures of central tendency and dispersion. Categorical variables were arranged in frequency and percentage. We sued the chi-square test for comparative analysis between subgroups. To analyze the time to oncological events, Kaplan-Meier curves were made. The study of similarity and causality between the results was conducted with the Log-rank test. Values of p<0.05 were considered statistically significant. Statistical analysis was performed using the Stata/SS software, version 14.1 (StataCorp LP, United States).

The project was approved by the Ethics in Research Committee of the Federal University under number 510304.7.0000.0096.

### Lymph node marking and surgery

**Figure 1:**
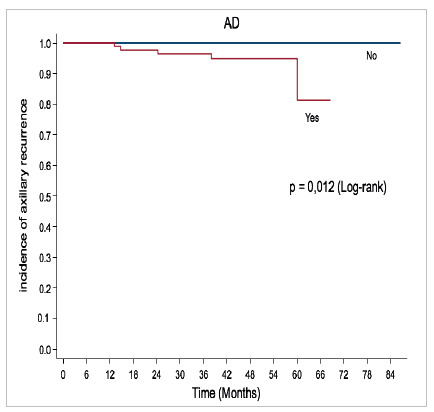
Axillary recurrence according to axillary preservation or dissection. AD = axillary dissection.

Prior to neoadjuvant therapy, the patients underwent ultrasonographic evaluation of the axilla on the same side of the tumor. When any suspicious lymph node was identified during this evaluation, fine-needle aspiration was performed to collect cytological material (Figure 1). In the case of identification of more than one suspicious lymph node, we prioritized the evaluation of the largest diameter lymph node. In the same intervention, we performed peri-lymph node marking using a 4% carbon suspension.

**Figure 2:**
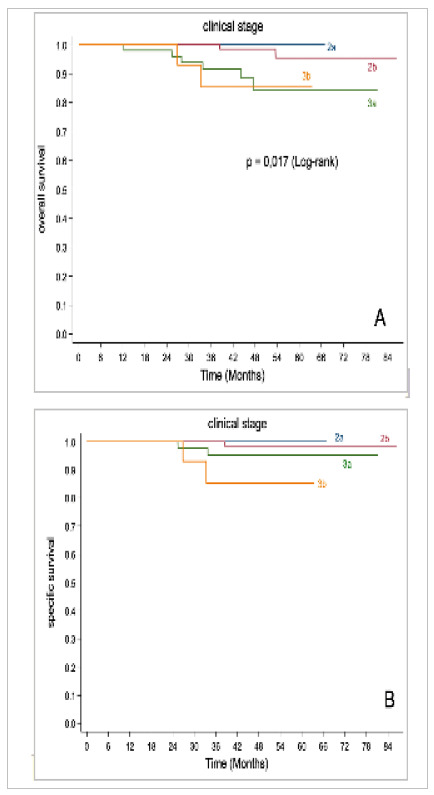
Survival and clinical stage, A: overall survival; B: specific survival.

After completion of systemic treatment, the patients underwent a reassessment to determine the extent of locoregional response to treatment, and subsequently underwent surgical intervention. During the surgical procedure, after the administration of anesthesia, we injected 1-2ml of Patent Blue Dye V in the periareolar region of the breast, followed by an approach to the armpit. During this procedure, we identified and removed lymph nodes stained with patent blue dye, as well as those previously marked with 4% carbon suspension (Figure 2).

During surgery, we conducted a pathological evaluation by freezing, and thus the patients were directed to axillary emptying or not. If the intraoperative pathological result was positive, indicating the presence of cancer cells, lymphadenectomy was performed, with the removal of at least 10 lymph nodes from the axillary chain, visualizing the anatomical structures that limit Berg’s levels 1 and 2. On the other hand, if the result was negative, indicating the absence of cancer cells, the intervention in the armpit was terminated.

After the surgical intervention, the specimens were immersed in vials containing 10% formaldehyde, where they remained for a period of more than 24 hours. Then, they were sectioned along their longitudinal axis into slices of up to 2 mm thickness and processed for histological analysis, including microtomy (with a 4-micrometer cut) and staining with hematoxylin and eosin.

For the cytological analysis prior to the systemic neoadjuvant treatment, the material obtained through fine needle aspiration puncture (FNA) was deposited on slides, dried in air, and then stained by the May-Grünwald-Giemsa method, being covered with coverslips. The analyses were performed using a Nikon Eclipse E200 microscope (Nikon Corporation, Tokyo, Japan) at magnifications of 40x, 100x and 400x. This process allowed a detailed evaluation of the morphological and cytological characteristics of the samples, contributing to a more accurate understanding of the condition of the tissues and cells studied.

### Follow-up

We gathered relevant data for statistical analysis from the medical records on the clinical stage, as defined by the TNM28 system, as well as on the tumor and axillary anatomopathological classification, in addition to the immunohistochemistry results.

We collected data on the axillary stage at two moments: before neoadjuvant systemic therapy, by means of imaging tests, categorized with the prefix “c”; and after surgery and systemic therapy, through the result of the final pathology, categorized with the prefix “yp”.

For a precise correlation between elapsed time and oncological outcomes, we recorded the dates of surgery, of event of interest, and of last visit. Follow-up was done through two medical consultations per year for the first two years and one annual consultation until ten years from the date of surgery.

## RESULTS

Between July 2014 and January 2019, 181 women underwent axillary marking prior to neoadjuvant treatment. We excluded five patients due to lack of adequate data and eight due to loss to follow-up. The final number of patients analyzed was 176 for lymph node identification in the two methods employed and 168 for oncological outcomes. Six individuals did not have their immunohistochemistry data categorized. The demographic characteristics of the study cohort are presented in Table 1.

**Table 1:** Distribution of clinical, pathological, and surgical characteristics..

Variable	Valid N	Classification	n	%
T	176	1	18	10,2%
		2	97	55,1%
		3	49	27,8%
		4	12	6,8%
N	176	1	152	86,4%
		2	24	13,6%
Clinical stage	176	2nd	17	9,7%
		2b	90	51,1%
		3rd	55	31,3%
		3b	14	8,0%
Histological Type	176	Ductal	141	80,1%
		Lobular	12	6,8%
		Duct lobular	7	4,0%
		Mucinous	7	4,0%
		Micropapillary	3	1,7%
		Apocrine	2	1,1%
		Medullary	2	1,1%
		Metaplastic	2	1,1%
RE	170	Negative	45	26,5%
		Positive	125	73,5%
RP	170	Negative	56	32,9%
		Positive	114	67,1%
HER2	170	Negative	129	75,9%
		Positive	41	24,1%
Variable	Valid N	Classification	n	%
Positive FNA	176	No	63	35,7%
		Yes	113	64,2%
Axillary dissection	176	No	89	50,6%
		Yes	87	49,4%

Regarding axillary dissection, this procedure was omitted in 89 cases (50.6%), while clinical suspicions were confirmed in 113 cases (64.2%) by FNA.

Events analyzed included local recurrence (LR), axillary recurrence (AR), distant disease-free survival (DDFS), overall survival (OS), and breast cancer specific survival (SS). We excluded four patients from the analysis due to the lack of data on breast cancer deaths on their death certificates.

We included 176 patients in the analysis of the detection rate of the carbon-labeled lymph nodes and the sentinel lymph node. The sentinel lymph node (SLN) detection method was unsuccessful in 31 (17.6%) cases, while the carbon-labeled lymph node (CLLN) was not identified in eight (4.5%) procedures. We observed coincidence between the sentinel and carbon-labeled lymph nodes in 93 of 176 (52.8%) patients, whereas there was no coincidence in 44 (25%) cases. At least one of the methods was not identified in 39 (22.1%) procedures. None of the surgeries had no lymph node identified by both methods. The lymph node identification rate according to the method is shown in Table 2.

**Table 2:** Lymph node identification rate by the methods applied.

Variable	Valid N	Classification	n	%
Positive SLN	176	No	101	57,4%
		Yes	44	25,0%
		Not found	31	17,6%
Positive SLN (pooled)	176	No	101	57,4%
		Yes/Not Found	75	42,6%
Positive CLLN	176	No	109	61,9%
		Yes	59	33,5%
		Not found	8	4,5%
Positive CLLN (pooled)	176	No	109	61,9%
		Yes/Not Found	67	38,1%
Coincident lymph nodes	176	Not applicable	39	22,1%
by both methods		No	44	25%
		Yes	93	52,8%
Coincident lymph nodes	137	No	44	32,1%
by both methods (excluding non-applicable ones)		Yes	93	67,8%

Table 3 presents the frequency and percentages of cases according to the anatomopathological results of the SLN and the CLLN, categorizing the lymph nodes not found as positive in the “yes/not found” category and as negative in the “no” category for neoplasm-free lymph nodes. This grouping was carried out with the aim of evaluating the decision on axillary lymphadenectomy after pathological analysis, given that, in the absence of lymph nodes evaluated, axillary lymphadenectomy is indicated. When condensing the lymph nodes not found with the positive lymph nodes, the sample agreement was 148 in 176 cases (81.4%), with a Kappa agreement coefficient of 0.67 (95% CI 0.56 -0.78), indicating a good agreement.

**Table 3:** Frequency and percentages of anatomopathological results and methods of lymph node identification considering a node not found as positive.

Positive SLN	Positive CLLN		Total
	No	Yes	
No	91	10	101
	51,7%	5,7%	
Yes	18	57	75
	10,2%	32,4%	
Total	109	67	176

The distribution of oncological outcomes, percentages, and the mean and median duration of follow-up are detailed in Table 4. In the group of patients examined, with a median follow-up time of 49 months, we observed a statistically significant association between axillary dissection and the incidence of axillary recurrence (0/85 0% vs. 5/83 6%, p=0.012). Graph 1 shows the evolution of axillary recurrence over time, comparing individuals who underwent axillary lymphadenectomy and those who did not. We identified seven local recurrence (LR) events in 168 patients analyzed, corresponding to a rate of 4.1%.

**Table 4:** Oncological outcomes and times measures of central tendency.

Outcome	Classification	n	Follow-up (months)	
			Mean ± standard deviation	Median (range; IQR)
Local recurrence	No	161	49 ± 12	49 (24 - 87; 19)
	Yes	7	43 ± 11	40 (31 - 62; 17)
Axillary recurrence	No	163	49 ± 12	49 (24 - 87; 19)
	Yes	5	30 ± 19	24 (13 - 60; 23)
Distance recurrence	No	140	49 ± 12	49 (24 - 87; 19)
	Yes	28	24 ± 15	24 (0,9 - 51; 22)
Specific death	No	158	49 ± 12	49 (12 - 87; 19)
	Yes	5	32 ± 5	33 (25 - 38; 7)
Death	No	158	49 ± 12	49 (24 - 87; 19)
	Yes	10	34 ± 12	34 (12 - 54; 17)
Overall		168	49 ± 12	49 (12 - 87; 19)

The most prevalent oncological outcome was distant recurrence. DFS was observed in 140 of 168 patients (83.3%). The most frequent site of recurrence was bone, occurring in nine of 28 cases (32.1%), followed by the lung, present in seven of 28 cases (25%). We identified a significant association between distant recurrence and involvement of at least one lymph node after neoadjuvant therapy (11.3% vs. 23.9%, p=0.027), as well as between distant recurrence and axillary dissection (10.5% vs. 22.9%, p=0.025).

During follow-up, there were 10 deaths among the 168 patients analyzed, of which five were secondary to breast carcinoma and five had an undetermined cause. The analysis of the clinical stage proved to be the main factor related to lower OS and SS, with statistical significance (log-rank test, p<0.05), as illustrated in Graph 2. In addition, we observed an association between a decrease in OS in women who underwent lymphadenectomy compared with those who did not (97.6% vs. 90.4%, p=0.035), as shown in Table 5. 

**Table 5:** Association between death and prognostic factors.

Variable	Classification	n	death (%)	p*
Age (years)	<50	88	5 (5,7%)	
	≥50	80	5 (6,3%)	0,867
Clinical stage	2nd	17	0 (0%)	
	2b	87	2 (2,3%)	
	3rd	50	6 (12%)	
	3b	14	2 (14,3%)	0,017
Subtype	Luminal HER	26	1 (3,8%)	
	Luminal	93	5 (5,4%)	
	HER 2 super expression	14	1 (7,1%)	
	Threefold-	29	3 (10,3%)	0,414
Positive FNA	No	61	3 (4,9%)	
	Yes	107	7 (6,5%)	0,694
Axillary Dissection	No	85	2 (2,4%)	
	Yes	83	8 (9,6%)	0,035

[Table t6]

**Table 6 t6:** Associação entre status axilar pré terapia sistêmica e desfechos oncológicos.

Clinical Axillary Status	Outcome	Classification	n (%)	p*
cN1	Axillary recurrence	No	144 (98)	0,078
		Yes	3 (2)	
cN2		No	19 (90,5)	
		Yes	2 (9,5)	
cN1	Distant recurrence	No	127 (86,4)	0,003
		Yes	20 (13,6)	
cN2		No	13 (62,9)	
		Yes	8 (38,1)	
cN1	Death	No	140 (95,2)	0,069
		Yes	7 (4,8)	
cN2		No	18 (85,7)	
		Yes	3 (14,3)	

The difficulties in locating and obtaining death certificates, together with the insufficiency of information when finally found, became a considerable obstacle to determining the causes of death not recorded in the study, which significantly compromised the analysis of the outcome of specific overall survival.

In the pre-NST clinical classification, 147 (87.5%) women were categorized as cN1 and 21 (12.5%) as cN2, after excluding the patients due to loss to follow-up. In the cN1 subgroup, the occurrence of the most relevant oncological outcomes was three (2%) for AR, 127 (86.4%) for DFS, and 140 (95.2%) for OS. When comparing the main oncological outcomes between the cN1 and cN2 subgroups, we observed a significant association in the DFS outcome, favoring the cN1 group (86.4% vs. 62.9%, p<0.05).

## DISCUSSION

We present the application of carbon as a marker for axillary lymph nodes before NST and its association with sentinel lymph nodes, aiming to improve the rates of false negatives in evaluations after neoadjuvant systemic therapy, following the approach adopted in the Target Axillary Dissection Study^15^. In addition, our study reveals the oncological outcomes in a mean follow-up period of 49 months in women undergoing selective axillary dissection using 4% carbon as a marker, presenting a pioneering contribution to this field of research.

We selected carbon as a material for lymph node marking due to its characteristics of being static, non-absorbable, and inert, making it suitable for the intended purpose, since it is not susceptible to migration^29^. In addition to its technical advantages, there are also associated economic benefits. Marking for non-palpable lesions with carbon is more economical compared with metallic wire when performed in the same biopsy procedure^30^. This advantage becomes even more evident when compared with metal clip, with an approximate cost of US$ 70 (US$ 60 for ultrasound-guided biopsy and marking procedure, and US$ 10 for 4% carbon) in the Brazilian context^23^. In addition, carbon offers an alternative to the use of I125 seed, which presents potential risks related to radiation exposure and requires specialized equipment and trained personnel for implantation and removal^31^. Like the metal clip, the seeds may shift slightly after implantation, which may make it difficult to locate the lesion during surgery^32^ ^-^ ^35^. The rate of identification of carbon as a marker in our study was 95.4%, higher than the rate of metal clip^6^ ^,^ ^15^.

We observed higher distant recurrence in patients with greater pre-NST axillary involvement (13.6% vs. 38.1% - p<0.05) when comparing cN1 with cN2. It is important to note that correct staging before the start of treatment is an important tool in determining prognosis, in addition to immunohistochemistry, histological grade, and genomic platform28, although post-NST involvement is important in the choice of adjuvant treatment^11^ ^,^ ^12^.

Axillary recurrence is an uncommon outcome and, therefore, should not be considered as the primary event in the analysis of axillary management. In patients with negative axillary status or lymph node micrometastases, the axillary recurrence rate at a 42-month follow-up is only 0.7%^36^, being more frequent in the first 24 months of follow-up^37^ ^,^ ^38^. It is relevant to note that women who underwent axillary lymphadenectomy often have worse prognosis^28^ ^,^ ^38^ ^-^ ^40^, 6% of our sample submitted to lymphadenectomy had regional recurrence, while there was no AR in the group that underwent conservative axillary surgery. This is because the number of lymph nodes affected is inversely associated with breast cancer-specific survival (SS) and overall survival (OS), with the persistence of regional disease being the main reason for lymphadenectomy. 

The decision about performing axillary dissection can be improved by lymph node labeling prior to neoadjuvant chemotherapy. The lack of agreement between the carbon-labeled lymph node and the sentinel lymph node does not necessarily alter the decision on axillary lymphadenectomy. For example, even if they do not match, both lymph nodes may be positive, leading to the decision to evacuate the axilla. We observed a rate of agreement in the decision to axillary dissection in 148 out of 176 cases (81.4%), with a Kappa coefficient of 0.67 (95% CI 0.56-0.78), indicating a good agreement when considering the broad decision. However, the high rate of SLN non-identification, 17.6% in this cohort, can be attenuated with pre-systemic therapy marking.

This study has some limitations. The sample is not homogeneous, since some patients underwent axillary dissection due to preoperative clinical suspicion that was not confirmed in the anatomopathological examination. The small number of patients limits the evaluation of subgroups. In addition, it lacked a control group according to the criteria of the ACOZOG Z1071 study, i.e., with double labeling and removal of at least three lymph nodes^6^. Moreover, post-neoadjuvant therapies were not performed at the time of study recruitment, so one of the doubts about the benefit of reducing false negatives in the axillary evaluation cannot be tested.

The addition of other drugs after neoadjuvant therapy has become the target of research and, when selecting patients who need more therapy, one chooses those who did not have a complete pathological response due to the worse prognosis in this scenario^41^ ^-^ ^44^. As a result, the use of PARP^45^ inhibitors and CDK 4/6^13^ inhibitors is already part of the arsenal of therapies that can be individualized for patients without a complete pathologic response to the treatment of early breast cancer. New studies should be designed to evaluate if the reduction of false negatives in axillary evaluation after selective axillary dissection has an impact on these patients.

## CONCLUSION

The use of carbon as a marker in selective axillary dissection is a reliable, low-cost, and easily identifiable material in 95.4% of axillary surgeries. The rate of AR in patients submitted to pre-systemic therapy 4% carbon labeling at 49 months of follow-up was 2.9%. Among oncological events, AR should not be used for axillary evaluation analysis after downstaging, since it is a rare event and is not necessarily related to OS or DDFS.
